# The development of career planning scale for junior high school students based on cognitive information processing theory

**DOI:** 10.3389/fpsyg.2023.1106624

**Published:** 2023-05-12

**Authors:** Peng Wang, Tiantian Li, Zhigang Wu, Xiao Wang, Jihao Jing, Jianjun Xin, Xiuchun Sang, Binrong Dai

**Affiliations:** ^1^School of Psychology, Shandong Normal University, Jinan, China; ^2^Faculty of Education, Shandong Normal University, Jinan, China; ^3^Preschool Education Department, Weifang Institute of Technology, Weifang, China; ^4^Shouguang City Luocheng Street to Liulu Experimental Primary School, Weifang, China; ^5^Jiangsu Provincial Key Constructive Laboratory for Big Data of Psychology and Cognitive Science, Yancheng Teachers University, Yancheng, China

**Keywords:** career planning, information technology course, scale developmenty, psychometric properties, cognitive information processing theory

## Abstract

Based on the career theory of Cognitive Information Processing (CIP), we selected scale items from literature reviews and expert guidance. The scale consisted of 28 items with 4 factors (interests, abilities, values, personality). To test the scale’s factor structure, we used confirmatory factor analysis (CFA), and the model was modified according to CFA results. The second-order confirmatory factor analysis was applied to the model of the scale to prove the rationality of the total score. The internal consistency were evaluated using Cronbach’s alpha coefficients. In addition, the composite reliability (CR) and average variance extraction (AVE) of the scale were also calculated to test the convergent validity. After related analyses, the scale was proved to have good psychometric properties, which can be used to measure junior high school students’ career planning level in information technology course from the aspects of interest, ability, values, and personality. The effect of the first-order confirmatory factor analysis model constructed in this study is not ideal. Therefore, on this basis, a second-order confirmatory factor analysis model is constructed in combination with existing literatures, and the rationality of the model is verified through data, which highlights the novelty of this study.

## Introduction

It is necessary to carry out career planning education and measure the level of career planning in primary and secondary schools. Because in China, the current educational philosophy holds that the starting point for students’ career planning should be during university education, and students are offered career planning courses when they face problems obtaining employment. However, for students at this stage, the study time of career planning is too tight to improve the level of career planning, and it cannot play a corresponding auxiliary role in the process of students seeking jobs. Therefore, career education should start from the more basic grade. It is very necessary to carry out formal and orderly career education to students in the process of their growth or to carry out conscious career education penetration to students in other disciplines (Bardick et al., 2006). As Chinese students study many subjects, there are many options for penetrating their vocational education, among which information technology courses are a good choice. However, there is no systematic and authoritative career planning test scale for information technology in China at present. Therefore, this study takes this as a starting point to develop and verify the scale in order to measure the career planning level of junior middle school students in information technology courses and improve the level of students’ career planning.

Chinese college students’ career planning education originates from the Western career guidance theory system. Since the introduction of higher education into China, college students’ career planning education has been rapidly applied to the employment education of Chinese college students, providing corresponding guidance and planning for college students who are about to prepare for employment (Fouad et al., 2016). College students are the most concerned people in Chinese career education at present (Guo and Jiang, 2019). However, students’ career planning should in fact be explored in middle school. While improving students’ comprehensive quality, career planning can help them find their favored career direction. When choosing a university, students can choose a suitable major according to their future career plans, purposefully improve their professional knowledge, and solve their employment problems to a certain extent. In secondary education, junior high school information technology teaching can successfully include career planning content, helping students to understand themselves, find their own interests, and exercise professional awareness.

Cognitive information processing (CIP) here refers to the vast area inside cognitive psychology that approaches human perception from the standpoint that a human is an active information processing participant (Lloyd and Jankowski, 1999). CIP theory can be used by itself or to facilitate the application of other career development theories and resources (Clemens and Milsom, 2008). Some scholars conducted a case study based on the theory of CIP, observed and interviewed the research subject, and gave certain guidance to his career development (Clemens and Milsom, 2008; Buzzetta et al., 2017). The final results show that CIP theory has certain positive effects on individual career planning and development (Reardon and Wright, 1999; Kato, 2020).

The information technology education is to provide students with relevant and rigorous educational experiences to enhance their career awareness in the field of information technology (IT or STEM technology; Hollman et al., 2019). Critical thinking and reasoning, as well as other soft skills such as teamwork and communication, which are emphasized in information technology course, are desirable attributes in any professional career field, but are especially important in computer and information science majors (Lou et al., 2011; Maltese and Tai, 2011). In addition to soft skills, hands-on problem-based learning and hands-on practice provide students with an opportunity to actively explore and potentially increase their interest in STEM and IT careers (Hollman et al., 2019). These abilities, which are emphasized in information technology course, are important to students as they move into adulthood and often serve as predictors of career and academic success. Students are more likely to select STEM and IT majors after they start college, because the relevant knowledge and skills they learn in IT courses enhance their self-efficacy in the field of IT (Hall and Michael, 2005). Therefore, it can be seen that information technology course are a good source of vocational information and can cultivate students’ skills and qualities related to future employment.

Career exploration is important for teenagers as they begin to explore themselves and explore potential career options at this time (Julien, 1999), and for teenagers, the decision-making and job exploration processes can be particularly stressful (Taveira et al., 1998). Proper career guidance for adolescents is necessary, and a useful approach is to give adolescents vocational knowledge in the courses they study. According to Larson and Majors, teenagers’ affective discomfort related to professional decision-making may be adaptive since it enhances their willingness to seek assistance, reducing the likelihood that they will make ill-informed judgments (Larson and Majors, 1998). Teenagers’ stress levels during job exploration and decision-making may be decreased by career planning (Witko et al., 2009).

In order to understand the level of career planning of junior high school students, this study developed a student career planning scale suitable for junior high school information technology course. The purpose of this study was to (a) develop a career planning level scale for use by junior high school students taking information technology course; and (b) provide students with targeted career guidance according to the test results to improve their level of awareness of the vocational application of their information technology course.

As far as we know, there are relatively few scales based on CIP-based career theory development to test the career planning level of students in junior high school information technology courses. This research fills this gap, which is where the innovation of this paper lies. In addition, helping students in junior high school information technology course understand their career plans is also conducive to stimulating their learning interest and motivation, and is also of great significance in improving academic performance and career planning levels.

## Literature review

### Career planning

Super first used the academic term “career” in his book *The Psychology of Careers* in 1957, in which he proposed that “career is the process of all positions that a person experiences in one’s life (p. 20).” He discussed the twelve basic propositions and five development periods of career development, thus laying the foundation for the theoretical framework of career development (Dietrich and Kracke, 2009). Rothwell proposed that career planning refers to individuals analyzing their own attributes and their relationship with the social environment to determine the direction, time, and program of action needed in order to achieve career goals (Rothwell, 1984).

Mark put forward the theory of career construction, the notion of “subjective career,” and advocated individual self-life design (Savickas, 2012). Later, in order to adapt to the development of society and the world economy, and to better provide students with professional theoretical and technical guidance, the United States began to implement career and technical education, and established a mechanism for mutual cooperation between schools and society to achieve a linkage between secondary and post-secondary education and vocational education, which is conducive to students’ employment or further study (Career, I. A. P. O. T. N. A. O. & Education, T, 2014).

Different theories of career development suggest that planning is important when facing career challenges (Savickas, 2005). Super indicated that the critical years for career preparation are late adolescence and early adulthood (Super, 1980).

According to Erickson, junior high school adolescents move into a stage of life were discovering their professional interests and seeking out career information is a crucial developmental step (Kracke, 1997). According to a more recent study by Bregman and Killen, young adults and adolescents favor making responsible career decisions that foster personal growth and disapprove of self-indulgent decisions centered on immediate objectives (Bregman and Killen, 1999). Adolescence is a crucial time for career planning since this is the time when people start to discover their skills, values, interests, and possibilities in order to prepare for career exploration (Dupont, 1991). Students are concerned about their future and want to improve their self-management and interpersonal skills in order to achieve their future job aspirations, according to Celotta and Jacobs’ research (Celotta and Jacobs, 1982). This understanding of the value of career planning appears to result from particular developmental phases.

While career planning is important, teenagers often encounter several obstacles in the career planning process (Pyne et al., 2002). Lack of information, lack of preparation, inaccurate information, discouragement in seeking information, a lack of financial resources, a lack of communication or self-confidence, and external or internal conflict are a few of these (Gati and Saka, 2001). Therefore, Hiebert advised that thorough assessments of students’ requirements should be the starting point of all efficient and complete guidance and counseling programs. Making the process more relevant to the kids and ensuring their genuine needs are met, which may be achieved by listening to them directly rather than assuming what they want and need based on adult prejudices and worries (Hiebert et al., 2001). A review of the literature revealed the significance of career planning for teenagers.

In order to influence future career planning and engage students in career planning activities that can have a significant impact on their lives, it is critical to examine junior high students’ perceptions of and needs for career planning. In the existing questionnaires on career planning level, researchers have compiled the questionnaires from different dimensions, and the focus of the questionnaires are different. For example, McAuliffe et al. developed and verified the career planning Confidence Level scale in their article, which consists of six dimensions, which are career decision readiness, self-assessment, generating options, information seeking, decision making and implementation decision (McAuliffe et al., 2006). Some researchers have also developed questionnaires containing 30 items in 6 dimensions, which are, respectively, goal setting, exploration, alternative detection, action plans and development activities (Shen, 2010). At present, there are few career planning scales available, especially for middle school students. Fantinelli et al. (2023) demonstrated the relationship between teenagers’ career choice and happiness. Through the observation and analysis of experimental subjects, they concluded that the influence of teenagers’ personal factors on career planning is closely related to their happiness in future life. Therefore, a good career education for teenagers is crucial to their future development.

Therefore, this study can enrich the research content and expand the research scope in the field of career planning, and form a career planning scale suitable for middle school students with certain innovation.

### Information technology course

Looking at it from China’s perspective, junior high school information technology course is a compulsory part of local (provincial) management course. Information technology course is practical course which pays attention to social reality, social needs, and social problems; is close to the real lives of students; and aims to help students solve practical problems. Students in junior high school must pass information technology course, which play an important role in the progress of students’ learning and social development. The core literacy of the information technology discipline consists of four aspects: information awareness, computational thinking, digital learning, and information responsibility (Huang, 2016). Judging from the development trend of information technology course worldwide, there has been a marked increase in the scientific and principled content taught in information technology course. For example, the information technology curriculum was at the core of literacy in the CSTA K-12 standards (2011 edition), the 2013 national curriculum for England and Wales planned to introduce computer science as an information technology teaching development (Furber, 2012), and in Australia in 2015, important teaching content was planned for a new course program that will calculate thought as the important content of the new information technology curriculum. In addition, the results of international research such as EU Core Literacy, American 21st Century Skills, the Horizon Report, as well as the concept and practice content of PISA, STEAM education, and Maker education, have all greatly emphasized the theoretical and scientific nature of information technology curriculum content (Wing, 2006). Besides, in 2015, the U.S. Congress passed the STEM Education Act, which officially included computer science in the STEM (science, Technology, Engineering, and Mathematics) curriculum, and since then, computer science have become part of the STEM curriculum (Guzdial and Morrison, 2016). STEM have great similarities with China’s local information technology course, for example, they both involve the teaching of computer science knowledge and the teaching of practical skills, and emphasize the cultivation of students’ practical ability. Both of these courses focus on preparing students for future development, employment and other aspects, and both emphasize the all-round development of students (Bybee, 2010). However, there are some differences between STEM education and information technology course in some aspects, which are specifically reflected in the fact that STEM education involves a wider range of subjects (Science, Technology, Engineering, and Mathematics) and the knowledge taught is more comprehensive, while information technology course focuses more on the computer knowledge and operation (Bacovic et al., 2022).

In China, in order for students to comprehend and master the fundamental knowledge and abilities of information technology, information technology course in primary and secondary schools need to inspire learners’ interests and attitudes toward learning. Students can make full use of information technology to solve the problems, such as information collection and processing, software operation and document processing, and the use of programming to solve real life problems, which would also improve their capacity for original thought (Liu and Su, 2018).

### The relationship between career planning and information technology course

In 1975, the American scholar Fintzy once pointed out that the continuous integration of careers education into discipline teaching is the most feasible and meaningful way to apply careers education (Fintzy, 1975). Information technology course is one of the sources of students’ career planning, and the integration of careers education into students’ courses is also one of the main ways to deliver careers education in China. As practical courses closely related to computers, information technology course enable students to experience a sense of what it is like to be employed in the computer industry in the process of learning (Bai, 2018). This gives them a deeper understanding of relevant majors and enables them to have a clearer future career plan.

In China, career and technical education is an important part of post-secondary education and an important channel to increase employment and promote economic development (Zhang et al., 2020). In general, the development of career and technical education is of constructive significance to both the country and individuals, which is why we pay attention to it. Education and teaching are not limited to classrooms, but usually are delivered through a combination of online and offline learning in classrooms and extracurricular activities. Career guidance is often provided to students through online teaching, requiring all students and teachers to use information technology in their classrooms, schools, communities, and families (Redmann and Kotrlik, 2004). In the process of integrating career planning into information technology course, some researchers have pointed out that vocational educators should integrate technology into the teaching process, applying technology to classroom teaching, and that they should research how to integrate the use of computer applications and the Internet into classroom teaching. Hollman emphasized the importance of highlighting IT in STEM education and exposing middle school students to IT so that students can not only prepare for success in STEM projects, but also for their future careers (Hollman et al., 2019). Chikahiko (2011) discussed the relationship between career planning and information technology education in terms of activities, goals, and content. In information technology education, learning topics about society and careers can promote students’ interest and attitudes toward the learning activities (Chikahiko, 2011). Vocational education has incorporated similar features into learning activities that are found in information technology education. In Korea, in order to provide students with better career education, a “Future and Career” teaching system that supports the use of computers and mobile devices has been developed (Song, 2014).

To sum up, combining the training objectives of careers education with information technology can not only cultivate students’ career planning ability, but also improve their information literacy, which will provide students with inspiration when it comes to their future career planning.

### Cognitive information processing model of career counseling

In order to better compile the scale, this study draws on the current relatively mature career theories, such as CIP theory.

CIP is a theory of career problem solving and decision making developed by a research team at Florida State University (Sampson et al., 2004). CIP theory is based on two essential elements: the pyramid of information processing, which contains three domains of information for effective career problem solving and decision making, and the five-step CASVE cycle, a career decision-making process that involves communication, analysis, synthesis, valuing, and execution (Buzzetta et al., 2017). A key tenet of CIP theory is that career problem solving and decision making are skills that can be learned, improved, and recalled for future career decisions (Sampson et al., 2004). The aim of CIP theory is to strengthen the control and planning of individual career by intervening the knowledge of individual career planning so as to develop their career planning ability accordingly.

**Figure 1:**
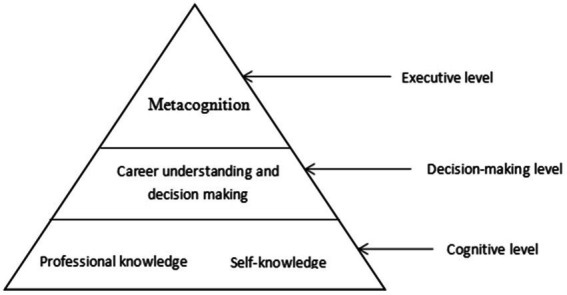
Career counseling information processing model diagram.

In the early 1980s, Peterson creatively applied CIP theory to career counseling for the leading research group in the field of cognitive psychology and created an information processing pyramid model at the information processing level (Aryee et al., 2007). The CIP theory offers a framework that integrates theory, practice, and research that may be applied to assist people in making wise career decisions (Sampson et al., 2011). As can be seen from the theoretical model diagram shown in Figure 1, the bottom layer of the model is the cognitive level, which includes the professional knowledge and self-knowledge required for information processing, and self-knowledge includes interests, abilities, values, and personality, etc. Professional knowledge recognizes work-related information, which is the cornerstone of the pyramid, and is also the basic component of information processing. At the middle level of the model is the decision level, where the information obtained is weighed to make career decisions. The top layer of the model is the executive level, which combines with metacognition to comprehensively monitor and reflect on career decisions.

More than 180 empirical studies have built on CIP theory and demonstrated its applicability in occupational intervention research (Wu, 2018). CIP theory has been successfully applied to a wide range of career services, including assessment, mentoring, and more. Its use in schools, career centers, and other organizations also shows its relevance to the topic (Toh and Sampson, 2021).

Therefore, designing a career planning assessment scale based on CIP theory can help students understand their career planning level and have a positive impact on students’ career decision-making status and skills, their knowledge of the next steps, and their sense of career experience.

### Summary of existing research

In the existing career planning measurement scales, the sample measured by this type of scales mostly concentrated in high schools, especially in colleges, and there are currently few career planning scales for junior high school students. In addition, existing career planning scales mostly measure the influencing factors of professional career planning, the mediating role of self-efficacy on students’ career planning, obstacles in students’ career planning, and the role of various teaching methods in vocational or university majors, etc., (Cardoso and Moreira, 2009; Fu et al., 2021; Chen et al., 2022; Fu et al., 2022).

Based on the above, this study focuses on designing a scale to measure the level of students’ career planning with the information technology course as the context and junior high school students as the research sample. To a certain extent, it is innovative, making up for the lack of current career planning scales for junior high school students and information technology course-specific backgrounds.

## Materials and methods

### Study design

#### Participants

In this study, we selected students from a middle school in Jinan, Shandong, China, and randomly selected 252 students (*M_age_* = 13.48, *SD* = 0.914, *N_boy_* = 126) to test the scale using cluster sampling method. These students come from different places with different backgrounds and can represent middle school students in Jinan. Their guardians signed a form to give consent for the students’ participation in the study. The research received ethical approval from the Ethics Board of the authors’ institution. Guardian of the children completed consent forms after being advised that the data they submitted was private and confidential. We certify that all participants in our study gave their consent for us to utilize their data for academic research and publication.

#### Data

Based on the career theory of CIP, this study assumes that a similar four-factor structure exists in the sample, with factor 1 as interest, factor 2 as competence, factor 3 as values, and factor 4 as personality. Scale items were obtained by combing past literature and combining with expert interviews. Since our study was based on CIP, like Bai, Dai et al., we conducted confirmatory factor analysis directly (Hang, 2020; Kato, 2020; Bai et al., 2021). In confirmatory factor analysis, if CFI > 0.8, TLI > 0.8, RMSEA<0.1, *χ*^2^/*df* < 3, the model fit was acceptable (Wen Zhonglin and Herbert, 2004). After the test, we collated the results and imported the structured data into IBM SPSS Statistics 26.0 and Mplus 8.3. The samples were randomly divided into two groups (sample A and sample B). Sample A consisted of 115 participants, while sample B consisted of 137 participants.

### Scale development

#### Identification of domain and item generation

The generation of items comes from three methods: (1) As the mature theoretical support, a review of the literature relating to CIP theory was carried out (Arrington, 2000; Creed et al., 2007); (2) we combined the characteristics of information technology course with the career planning scale, and developed a career planning scale suitable for information technology course; and (3) On the basis of CIP theory and existing tests, the initial version of this questionnaire was obtained. Under the guidance of a professor in the field of psychology and a teacher of information technology course, the pilot version of this questionnaire was formed. In the end, a 28-item questionnaire with four factors was organized into a frame including interests (Q1, Q8, Q15, Q16, Q18, Q21, Q25, Q28), abilities (Q2, Q9, Q12, Q14, Q17, Q23, Q27), values (Q3, Q4, Q6, Q13, Q19, Q20), and personality (Q5, Q7, Q10, Q11, Q22, Q24, Q26). A Likert five-point scoring method was used for all the items.

#### Psychometric properties

In order to verify the rationality of the generated items and the validity of the questionnaire, we carried out discrimination analysis, item-total analysis and confirmatory factor analysis, respectively. The career planning scale developed in this study is based on CIP theory. Because the four factors and the corresponding items are proposed before analyses, just like previous scholars, this study only conducted confirmatory factor analysis (Kato, 2020; Bai et al., 2021).

## Results

### Item discrimination

The sample results were divided into high group and low group according to the score of top 27% and bottom 27% (Lyu et al., 2020). As shown in Table 1, there were significant differences between high and low scores for each item (*p* < 0.05). Therefore, no items needed to be deleted at this stage.

**Table 1:** Item discrimination analysis.

Item	Group (M ± SD)		*t*
	Low grouping (*n* = 78)	High grouping (*n* = 79)	
Q1. In the information technology class, I am willing to take the initiative to learn information technology knowledge	3.50 ± 1.029	4.86 ± 0.549	10.359**
Q2. Through studying the information technology course, I can use a variety of tools and means to obtain information and solve information technology problems	3.50 ± 0.549	4.78 ± 0.335	12.433**
Q3. By studying information technology, I know that information technology is very relevant to my future	3.40 ± 0.972	4.89 ± 0.320	12.929**
Q4. I think the development of information technology will affect my own values	2.59 ± 1.012	4.25 ± 1.296	8.971**
Q5. By learning information technology, I will fully consider and finalize my choice before making a decision	3.14 ± 0.963	4.81 ± 0.579	13.178**
Q6. I think information technology is a practical and operational discipline; therefore, it must be taught in a rigorous and serious manner	3.62 ± 1.035	4.95 ± 0.221	11.203**
Q7. When the teacher asks a question related to information technology in class, even if I am not sure if my answer is correct, I will actively answer it	2.06 ± 0.779	4.05 ± 1.132	12.802**
Q8. I would like to know more about the work process and occupational characteristics of professionals, occupations, and industry personnel related to information technology	2.78 ± 0.921	4.86 ± 0.348	18.715**
Q9. In the process of making information technology projects work, I can plan the time reasonably and complete the information technology learning tasks as planned	3.19 ± 0.838	4.94 ± 0.254	17.743**
Q10. In the presentation of information technology class projects, I often reflect on my own characteristics through the feedback and evaluation of teachers and classmates	3.03 ± 0.953	4.84 ± 0.492	14.979**
Q11. Having taken an information technology course, I consider myself suitable for learning programming	2.45 ± 0,935	4.53 ± 0.769	15.285**
Q12. I think my teamwork ability, self-expression, and communication ability have been improved to some extent through studying the information technology course	2.85 ± 1.020	4.81 ± 0.573	14.857**
Q13. Through studying the information technology course, I realized that future social development is inextricably related to information technology	3.42 ± 0.987	4.96 ± 0.192	13.598**
Q14. I can use the skills I have learned in the information technology class to complete some work design	3.42 ± 0.890	4.92 ± 0.350	13.935**
Q15. Through studying the information technology course, I became interested in optional courses related to information technology offered by the school	2.58 ± 0.845	4.47 ± 0.889	13.656**
Q16. Through studying the information technology course, I began to imagine which majors would be more important to me in the future	2.88 ± 0.853	4.76 ± 0.625	15.733**
Q17. I can adopt novel methods to complete some challenging IT tasks	2.73 ± 0.989	4.82 ± 0.446	17.118**
Q18. I hope that the competitions related to information technology that I participate in in the future are what I love, rather than it being teachers or parents who help me make decisions	3.24 ± 1.047	4.85 ± 0.579	11.903**
Q19. Through the study of information technology, I consciously protect my personal privacy when surfing the Internet	3.69 ± 1.117	4.94 ± 0.462	8.741**
Q20. I think the information technology course will be helpful for my future life choices	3.18 ± 0.936	4.95 ± 0.273	16.126**
Q21. I believe that information technology plays an important role in future college majors and careers of interest	3.05 ± 1.005	4.91 ± 0.286	15.813**
Q22. I can discover the strengths and weaknesses of my character through the information technology course	2.92 ± 0.894	4.72 ± 0.576	15.003**
Q23. I can confidently present my IT work to my classmates	2.90 ± 0.975	4.78 ± 0.498	15.304**
Q24. By studying the information technology course, I can know whether my classmates’ personalities have great potential in this course	2.88 ± 0.882	4.81 ± 0.601	15.999**
Q25. Through learning information technology, I will discuss people and emerging technologies related to information technology with my classmates	2.63 ± 0.968	4.78 ± 0.547	17.207**
Q26. Through the study of information technology, I have a clearer understanding of my own personality	2.87 ± 0.958	4.59 ± 0.829	12.081**
Q27. I can divide my learning objectives of the information technology course reasonably and adjust them according to my own situation	2.95 ± 0.788	4.94 ± 0.245	21.404**
Q28. I think information technology is an important course. I am very interested in hot topics in the field of information technology and often collect information related to information technology	2.41 ± 0.829	3.72 ± 0.715	10.620**
Career planning	83.8718 ± 12.802	132.5945 ± 5.073	31.421**

**p < 0.01.

### The correlations between item and total score

The analysis of correlation between item and total score aims to examine the relationship between each item and the total score of the scale. Items that were not significantly correlated with the total score of the scale were not desirable and were therefore removed from the scale (Fu et al., 2021). After analysis, the correlation coefficients (0.505–0.867) between each item and the total score were significant; therefore, the scale has good item-total correlations and no items needed to be deleted at this stage.

### Confirmatory factor analysis

As mentioned above, because the four factors and the corresponding items are proposed before analyses, exploratory factor analysis was not performed, and this study only conducted confirmatory factor analysis (Kato, 2020, Bai et al., 2021).

#### First-order confirmatory factor analysis

**Figure 2:**
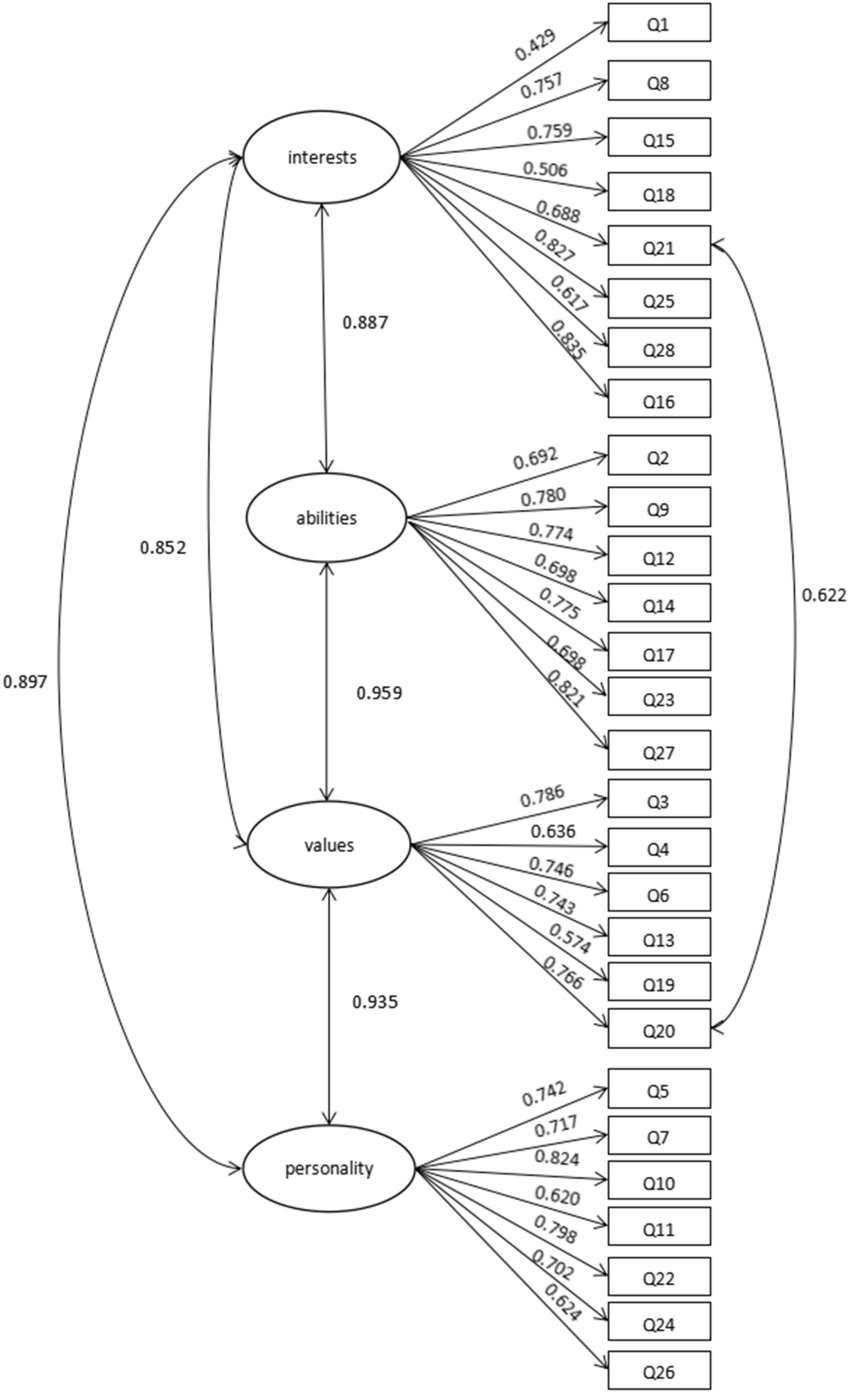
Four-factor model of first-order confirmatory factor analysis.

**Figure 3:**
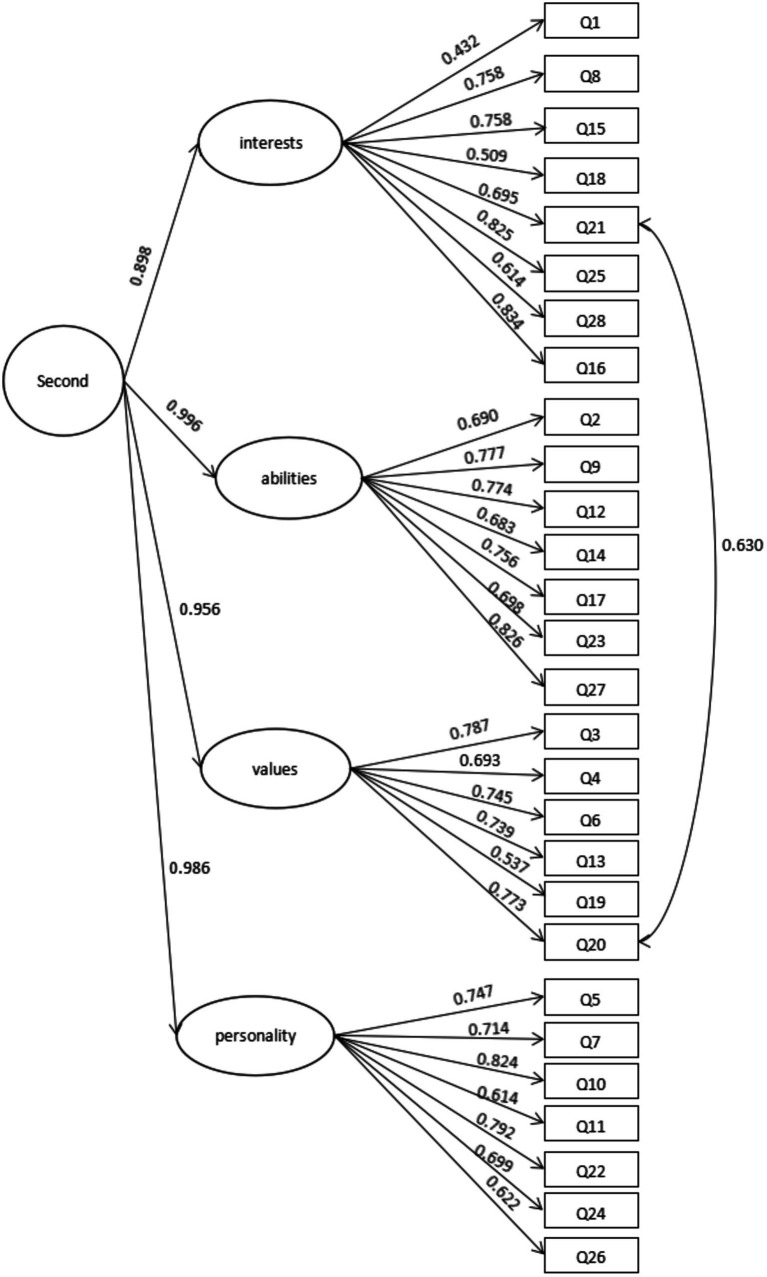
Four-factor model of second-order confirmatory factor analysis (*n* = 115).

When the CFA was conducted for the first time, we found that the indicators derived from the analysis showed that the original model (four factors, each containing seven items) did not fit well (*χ*^2^/*df* = 2.36, CFI = 0.797, TLI = 0.777, RMSEA = 0.109). According to the revised proposal, we made corresponding corrections to the original model, putting Q16 in factor 1, and linking Q20 with Q21, thus obtaining a new model. As illustrated in Figure 2, we conducted CFA on sample A (*n* = 115). The standardized factor loadings, which are depicted in Figure 3, exceeded 0.4 and ranged from 0.429 to 0.821; according to the standard value of the factor loading coefficient, if the factor loading coefficient is above 0.4, it is considered that the factor has good interpretability (Maskey et al., 2018). The results proved that there was a strong correlation between factors and items.

According to Wen Zhonglin and Herbert (2004), if RMESA is lower than 0.1, the model fits well. However, he also mentions that the fitting of the model does not depend on a single index (such as RMSEA). If a single index is close to the critical value and other indicators (such as CFI, TLI, etc.) meet the standards, the model can also be considered acceptable. After model revision, the first-order CFA assessed the correlation coefficients and factor loadings between the four factors and 28 items, as shown in Table 2. Among them, RMSEA was slightly higher than the standard indicator, and the other values were within the range of the standard indicator.

**Table 2:** Four-factor indices of first-order confirmatory factor analysis (*n* = 115).

Common index	*χ*^2^/*df*	CFI	TLI	RMSEA
Value	2.178	0.825	0.807	0.101 [0.091, 0.111]

#### Second-order confirmatory factor analysis

Based on the first-order CFA, we found that the correlation coefficient between the four factors was at a high level, so we speculated that there was a common high-order factor for these four factors. Therefore, on the basis of the first-order CFA, taking sample A (*n* = 115) as the subjects, a second-order CFA was conducted for this model, as shown in Figure 3. Compared to the initial model, we made the following adjustments in the second order model. It is composed of question 20, “I think the information technology course will be helpful for my future life choices,” and question 21, “I believe that information technology plays an important role in future college majors and careers of interest. “Information technology plays an important role in future college majors and careers of interest.” Both emphasize the role of information technology course in the development of students’ future careers. Therefore, these two topics are highly relevant and it is reasonable to associate them. Question 16, “Through studying the information technology course, I began to imagine which majors would be more important to me in the future.” Its purpose is to explore students’ learning interests and future interest majors, so it is included in factor 1 (interest).

As shown in Figure 3, there was a high-order factor that was closely related to the four factors (interest, ability, values, and personality) in the first-order model, and the correlations between high-order factors and the four factors in the first-order model were very good (all above 0.8). In addition, the factor loading coefficients between the four factors and each item also increased to some extent. In the second-order CFA, as shown in Table 3, these indices met the criteria, so we can draw the conclusion that the model has a good fitting index.

**Table 3:** Four-factor indices of second-order confirmatory factor analysis (*n* = 115).

Common index	*χ*^2^/*df*	CFI	TLI	RMSEA
Value	2.168	0.826	0.809	0.100 [0.091, 0.110]

### Scale re-evaluation

From the correlation matrix among the various dimensions listed in Table 4, it can be seen that the four dimensions of the career planning scale for junior high school students are correlated with each other, all reaching above 0.775. This result shows that there is a strong relationship between the dimensions, so we directly performed a second-order confirmatory factor analysis.

**Table 4:** The correlation matrix among the various dimensions.

		Interests	Abilities	Values	Personality
Interests	Pearson correlation	1	0.792**	0.775**	0.815**
	Sig. (two-tailed)		0.000	0.000	0.000
	Cases	115	115	115	115
Abilities	Pearson correlation	0.792**	1	0.832**	0.859**
	Sig. (two-tailed)	0.000		0.000	0.000
	Cases	115	115	115	115
Values	Pearson correlation	0.775**	0.832**	1	0.781**
	Sig. (two-tailed)	0.000	0.000		0.000
	Cases	115	115	115	115
Personality	Pearson correlation	0.815**	0.859**	0.781**	1
	Sig. (two-tailed)	0.000	0.000	0.000	
	Cases	115	115	115	115

** Correlation is significant at the 0.01 level (two-tailed).

### Construct validity

**Figure 4:**
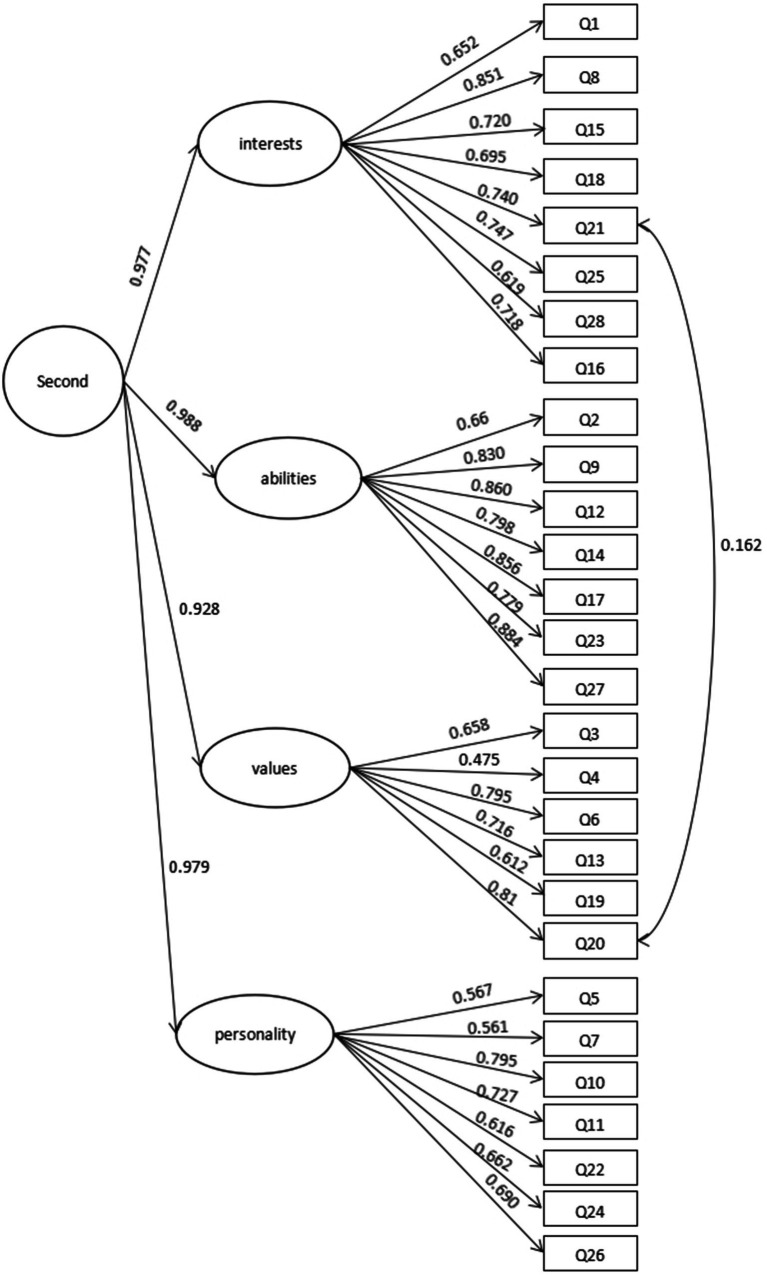
Four-factor model of second-order confirmatory factor analysis (*n* = 137).

In order to verify the effectiveness of the second-order CFA, we conducted second-order CFA on sample B (*n* = 137) again. The results of the second-order CFA for sample B (Figure 4) show that the factor loadings between the four first-order factors and the items were all above 0.4, and the correlation coefficients between the second-order factors and the first-order factors were all above 0.9, which proves that the second-order model has a good structure (Wang et al., 2022). As shown in Table 5, the results show that the fitting indices of the model were good.

**Table 5:** Four-factor indices of second-order confirmatory factor analysis (*n* = 137).

Common index	*χ*^2^/*df*	CFI	TLI	RMSEA
Value	2.380	0.826	0.809	0.100 [0.092, 0.109]

### Reliability

#### Coefficients of internal consistency

After the final structure of the model was determined using CFA, the internal consistency was assessed using Cronbach’s alpha; Cronbach’s alpha value above 0.7 is generally considered acceptable, and 0.8 and above is considered desirable (Tavakol and Dennick, 2011; Taber, 2018). As shown in Table 6, α was 0.965, which shows the reliability of the scale is very high.

**Table 6:** Cronbach’s α coefficients.

Items	Alpha coefficient of deleted item	Cronbach’s α coefficient
Q1	0.965	0.965
Q2	0.964	
Q3	0.964	
Q4	0.966	
Q5	0.964	
Q6	0.964	
Q7	0.965	
Q8	0.963	
Q9	0.964	
Q10	0.963	
Q11	0.964	
Q12	0.963	
Q13	0.964	
Q14	0.964	
Q15	0.964	
Q16	0.964	
Q17	0.963	
Q18	0.965	
Q19	0.965	
Q20	0.964	
Q21	0.964	
Q22	0.964	
Q23	0.964	
Q24	0.964	
Q25	0.964	
Q26	0.965	
Q27	0.963	
Q28	0.965	

#### Reliability of dimensions

As shown in Table 7, Cronbach’s alpha coefficient of the four dimensions were greater than 0.8, indicating that each dimension has good internal consistency and high reliability (Arifin, 2018).

**Table 7:** Reliability coefficients of each dimension.

Dimensions	N	Cronbach’s α coefficient
Interests	8	0.891
Abilities	7	0.917
Values	6	0.836
Personality	7	0.886

#### Results of model AVE and CR indices

The CR value of the total structure was greater than 0.7, indicating composite reliability (Mirzaei et al., 2019). As shown in Table 8, the AVE values of the two dimensions were less than 0.5, but an AVE value greater than 0.4 is acceptable (Mirzaei et al., 2019). According to Fornell-Larcker’s previous research, convergent validity can be confirmed when the AVE value is less than 0.5 but the CR value is all above 0.6 (Fornell and Larcker, 1981). According to Table 7, convergent validity constructs were confirmed.

**Table 8:** Results of model AVE and CR indices.

Factor	Average variance extracted (AVE value)	Composite reliability (CR value)
Interests	0.520	0.896
Abilities	0.660	0.931
Values	0.481	0.840
Personality	0.415	0.830
Career planning	0.516	0.967

## Discussion

The results of this study indicate that the junior high school career planning scale based on CIP theory is an effective measuring tool to assess junior middle school students’ level of career planning in information technology. Career education teachers or psychological guidance teachers can use theory-based tools to evaluate students’ career plans or intentions in information technology course and make effective educational interventions to target groups based on the results (Solhi et al., 2012; Ebrahimipour et al., 2015).

Evaluation of construct validation is the most important step to determine the validity of the development (Mirzaei et al., 2019). Factor analysis is the best method to achieve this purpose (Shirvani et al., 2014).

CFA is used to test the correlation between factors and items and verify whether there is consistency between the data and theoretical structure (Brown, 2006; Mirzaei et al., 2019). CFA was conducted on the four factors and 28 items contained in the scale. The initial results showed that the scale had unsatisfactory structural validity. Therefore, the scale model was revised, with the exception of RMSEA, which was slightly higher than 0.1, other fit indexes of CFA all met the requirements, indicating that the model structure was good (Brown, 2006). Through first-order confirmatory factor analysis, we found that the first-order factors were highly correlated with each other, which indicated that the latent variables do not work perfectly as independent variables, and their correlations reflect the presence of more general structures in secondary-order hierarchies (Joseph et al., 2016). So, we believed that there was a common factor affecting the changes of the first-order four factors. To verify this conjecture, we carried out second-order confirmatory factor analysis on the model. This method attempts to obtain a more meaningful data collection method by assuming that the latent variables in the common variance belong to one or more higher-order factors and that the model have two levels when analyzing the data (Gatignon, 2014). The expected structure should first be produced by first-order factor analysis to determine the proper fit of the structure, making it convenient for second-order factor analysis to evaluate structural equation models (Anderson, 1998). Therefore, based on the first-order model, a second-order CFA was conducted for sample A and sample B, respectively. The application of second-order confirmatory factor analysis with sample A and sample B verifies the existence and rationality of second-order factor and proves that it is reasonable to calculate the total score of the test. In confirmatory factor analysis, it is important to use second-order confirmatory factor analysis to analyze the collected data, and justify the total scores of the questionnaire (Kulachai et al., 2021; Wang and Shengbin, 2021).

Reliability analysis of the scale was carried out in the process of compiling the scale. Cronbach’s α coefficients were used to judge the reliability and internal consistency of the scale (Fu et al., 2021). The results showed that the Cronbach’s α coefficients of the whole scale and all dimensions were above 0.9, indicating the high reliability and internal consistency of the scale.

Finally, in this study, the convergent validity was assessed. According to the Fornell-Larcker criterion, convergent validity was confirmed (Fornell and Larcker, 1981). As shown in Table 7, the CR values were all above 0.8 and AVE values were all above 0.4, indicating the high convergent validity of the scale, meeting the indicator requirements (Mirzaei et al., 2019).

There are several differences between this study and previous researches. Compared with traditional career planning scales, the scale developed in this study takes information technology as the specific course. This makes the use of the scale more targeted, enabling it to better measure and guide students’ career planning in the field of information technology. Based on CIP theory, this study fully considered various influencing factors of career development. According to CIP theory, four components are essential for making effective career decisions, which are self-knowledge, options knowledge, decision skills and executive skills. Executive processing skills emphasize the metacognitive processes of how one thinks about one’s career decisions and other areas (i.e., self-knowledge, choice knowledge and decision skills). This is the main theoretical source and basis of our questionnaire compilation (Osborn et al., 2020). Finally, this study changed the previous tradition of taking undergraduates and postgraduate students as the research subjects (Chikahiko, 2011; Lyu et al., 2020; Osborn et al., 2020). This makes the research novel and helps junior high school students to understand occupation-related knowledge, contact occupation classification and occupational characteristics, which can pave the way for their future career planning and is beneficial to the future development of students. In addition, this study compared with the past about career planning level of scale, the second-order factor are verified, and is able to prove the rationality of the total test scores and the validity of the questionnaire structure.

## Limitations

Although this study provides a measuring tool for the career planning of junior high school students taking information technology courses, there are still some limitations in the research process.

First of all, the sample of this study is limited with participants mainly recruited from a province in eastern China. Therefore, the results may not be universal in the case of large economic and cultural differences nationwide.

Second, the scale was produced in China, and the values dimension item in the scale is likely to be influenced by Chinese culture, so it is uncertain whether the measurement standard will change in different cultural contexts; therefore, it is necessary to apply relevant research designs in different cultural contexts to improve the application of the scale.

Finally, due to the influence of COVID-19 in recent years there have been employment difficulties. The emergence of the epidemic might have a certain influence on students’ career planning. This study did not treat it as a potential factor; future research needs to explore the potential impact of epidemics and other factors.

In addition, we will continue to collect evidences on criterion-related validity in subsequent studies.

## Conclusion

Based on CIP theory, we developed a career planning scale suitable for use in junior high school information technology course. After related analyses, the scale developed was shown to have good psychometric properties, which can be used to measure junior high school students’ career planning level in information technology course from the aspects of interest, ability, values, and personality. What’s more, this study proved that the existence of the second order model of the questionnaire, which means a common factor explains the variance of the four dimensions.

## Data availability statement

The raw data supporting the conclusions of this article will be made available by the authors, without undue reservation.

## Ethics statement

The studies involving human participants were reviewed and approved by Shandong Normal University. Written informed consent to participate in this study was provided by the participants’ legal guardian/next of kin.

## Author contributions

PW, TL, ZW, XW, JJ, JX, XS, and BD contributed to the study conception and design. XW, XS, and JX performed the material preparation, data collection and analysis. TL wrote the first draft of the manuscript. JJ contributed to drafting the literature review, discussion. PW, ZW, and BD as well as all authors commented on previous editions of the manuscript and the overall editing of the manuscript. All authors contributed to the article and approved the submitted version.

## Funding

This research is supported by the “Special Creation and Integration” Characteristic Demonstration Curriculum Construction Project of shandong Normal University in 2021 [grant no. SDNU2021ZCRH025], and the project of Jiangsu Pro vincial Key Constructive Laboratory for Big Data of Psychology and Cognitive Science [grant no. 72592062001G], and Weifang Institute of Technology 2022 annual university-level humanities and social science project [grant no. SK-X202203].

## Conflict of interest

The authors declare that the research was conducted in the absence of any commercial or financial relationships that could be construed as a potential conflict of interest.

## Publisher’s note

All claims expressed in this article are solely those of the authors and do not necessarily represent those of their affiliated organizations, or those of the publisher, the editors and the reviewers. Any product that may be evaluated in this article, or claim that may be made by its manufacturer, is not guaranteed or endorsed by the publisher.
